# Using an Endoluminal Functional Lumen Imaging Probe (EndoFLIP™) to Compare Pyloric Function in Patients with Gastroparesis to Patients After Esophagectomy

**DOI:** 10.1007/s11605-022-05502-x

**Published:** 2022-11-14

**Authors:** Florian Lorenz, Stefanie Brunner, Felix Berlth, Thomas Dratsch, Benjamin Babic, Hans Friedrich Fuchs, Thomas Schmidt, Erkan Celik, Daniel Pinto dos Santos, Peter Grimminger, Christiane Josephine Bruns, Tobias Goeser, Seung-Hun Chon

**Affiliations:** 1 Department of Gastroenterology and Hepatology, Interdisciplinary Endoscopy Unit, University Hospital of Cologne, Cologne, Germany; 2 Department of General, Visceral, Cancer and Transplant Surgery, University Hospital of Cologne, Kerpener Street 62, 50937 Cologne, Germany; 3 Department of General, Visceral and Transplantation Surgery, University Hospital of Mainz, Mainz, Germany; 4 Department of Diagnostic and Interventional Radiology, University Hospital of Cologne, Cologne, Germany; 5 Department of Radiology, University Hospital Frankfurt, Frankfurt, Germany

**Keywords:** EndoFLIP™, Pyloric distensibility, Esophagectomy, Delayed gastric conduit emptying, Gastroparesis

## Abstract

**Background:**

Gastroparesis (GP) occurs in patients after upper gastrointestinal surgery, in patients with diabetes or systemic sclerosis and in idiopathic GP patients. As pyloric dysfunction is considered one of the underlying mechanisms, measuring this mechanism with EndoFLIP™ can lead to a better understanding of the disease.

**Methods:**

Between November 2021 and March 2022, we performed a retrospective single-centre study of all patients who had non-surgical GP, post-surgical GP and no sign of GP after esophagectomy and who underwent our post-surgery follow-up program with surveillance endoscopies and further exams. EndoFLIP™ was used to perform measurements of the pylorus, and distensibility was measured at 40 ml, 45 ml and 50 ml balloon filling.

**Results:**

We included 66 patients, and successful application of the EndoFLIP™ was achieved in all interventions (*n* = 66, 100%). We identified 18 patients suffering from non-surgical GP, 23 patients suffering from GP after surgery and 25 patients without GP after esophagectomy. At 40, 45 and 50 ml balloon filling, the mean distensibility in gastroparetic patients was 8.2, 6.2 and 4.5 mm^2^/mmHg; 5.4, 5.1 and 4.7 mm^2^/mmHg in post-surgical patients suffering of GP; and 8.5, 7.6 and 6.3 mm^2^/mmHg in asymptomatic post-surgical patients. Differences between symptomatic and asymptomatic patients were significant.

**Conclusion:**

Measurement with EndoFLIP™ showed that asymptomatic post-surgery patients seem to have a higher pyloric distensibility. Pyloric distensibility and symptoms of GP seem to correspond.

## Introduction

Delayed gastric emptying in the absence of a mechanical obstruction is defined as gastroparesis (GP). GP can be caused by different pathomechanisms that lead to abnormal motility of the stomach. Several mechanisms can be distinguished; for example, impaired fundus relaxation due to increased fundic tone, decreased antral contractility and decreased pyloric relaxation.^1^

Different causes for these abnormalities have been identified. For example, the interstitial cells of Cajal (ICC), which act as a pacemaker of gastric motility, are often reduced in number and functionality. Moreover, in post-surgical delayed gastric emptying, a disruption of the vagal nerve can cause dysregulation of motility.^2^ While these are possible causes, the exact mechanisms of gastroparesis are not yet fully understood.

Varying symptoms like post-prandial fullness, bloating, abdominal pain, nausea, emesis and weight loss occur due to impaired gastric motility and cause a significant disease burden for gastroparesis patients. As the heterogeneity of the causes of gastroparesis impedes an effective therapy, new insights into the disease are crucial.^3^

**Fig. 1:**
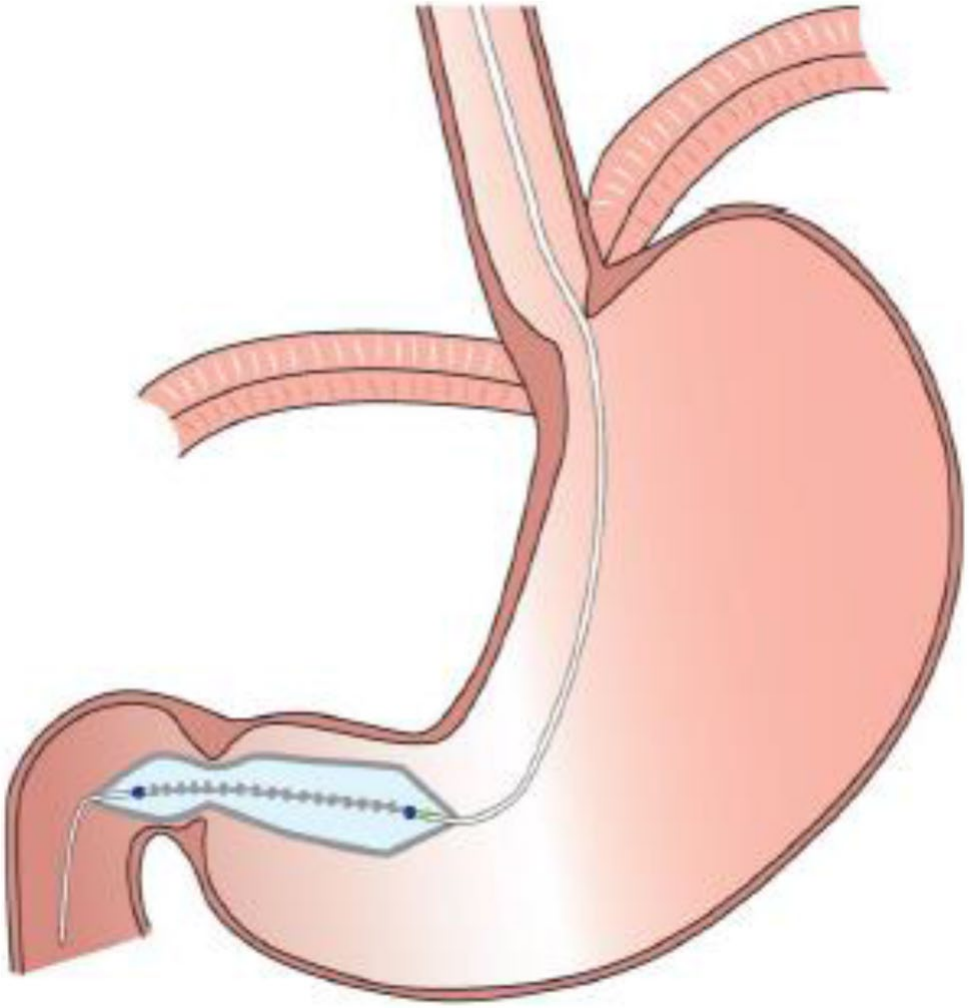
Placement of EndoFLIP™ throughout the pylorus

One novel endoscopic approach to evaluate the pylorus is an endoscopic functional luminal imaging probe (EndoFLIP™, Medtronic, Minneapolis, USA). It measures pressure, diameter and distensibility (DI) in order to study biomechanical properties of gastrointestinal sphincters (Fig. 1).^4^ In the past years, several studies have been performed to implement EndoFLIP™ measurement for the pylorus.^5,6^

However, only few available studies investigate the efficacy of EndoFLIP™ measurement of the pylorus in gastroparesis. The purpose of this study is to focus on the pyloric distensibility of a patient cohort categorized in three different subgroups: patients with GP without prior surgery, patients with GP after upper-GI surgery and asymptomatic post-surgical patients. Our goal was to evaluate the pyloric distensibility in these patient groups and to compare them.

## Material and Methods

### Patients

This retrospective study was conducted jointly at the Department of Gastroenterology and Hepatology and the Department of General, Visceral, Cancer and Transplant Surgery at the University Hospital Cologne. Data were retrieved from our prospectively maintained endoscopic database “Clinic WinData” (version 8.06; E&L medical system GmbH, Erlangen, Germany) and from our hospital database “Orbis” (version 08,043,101; Agfa HealthCare N.V., Belgium). The following information was collected: demographic and clinical patient characteristics, details of the disease and endoscopic findings (Table 1).

**Table 1:** Patients’ characteristics in the different groups

	**Non-surgical gastroparesis**	**Post-surgical gastroparesis**	**Post-surgical asymptomatic**
N	18	23	25
Age (mean)	51 (21–83)	65 (31–84)	60 (37–72)
Men/women	3/15	14/9	23/2
Cause of gastroparesis	9 idiopathic 5 diabetic 2 systemic sclerosis 1 Ehlers-Danlos syndrome 1 mucoviscidosis	5 fundoplication 18 esophagectomy	-
BMI (kg/m^2^) (mean)	23.4 (16.7–31.6)	26.8 (19.8–42.3)	25.5 (17.6–38.8)
Smoking status	15 non-smokers 2 active/former smokers 1 unknown	16 non-smokers 7 active/former smokers	12 non-smokers 13 active/former smokers
ASA (mean) [95% KI]	1.9 (1.6–2.2)	2.4 (2.1–2.7)	1.8 (1.5–2.1)
CCI (mean) [95% KI]	1.4 (0.8–2.0)	0.6 (0.3–0.9)	0.3 (0.0–0.6)
Retention of food in the endoscopy	16% (*N* = 3)	69% (*N* = 16)	20% (*N* = 5)

We included both the patients who received an esophagogastroduodenoscopy including EndoFLIP™ measurement due to delayed gastric emptying and those who received an EGD as part of the post-surgical routine after esophagectomy being asymptomatic and showing no sign of DGE. Delayed gastric conduit emptying in post-surgical patients was defined as delayed contrast passage in upper GI water-soluble contrast radiogram combined with characteristic symptoms of DGE. Water-soluble contrast radiogram was performed in an upright position using 100 ml of contrast medium and timed post-surgically as soon as symptoms of DGE occurred. Gastroparesis in non-surgical patients was defined as pathological gastric emptying scintigraphy combined with characteristic symptoms of DGE. Gastric emptying scintigraphy was performed with a solid meal containing a radioisotope using standard protocols with imaging after 0, 1, 2 and 4 h.^7^ No pyloric treatment such as pyloromyotomy, pyloroplasty or Botox injection was performed prior to our analysis.

Between November 2021 and March 2022, 66 patients underwent EndoFLIP™ measurement. All patients underwent a standardized EGD with photo and video documentation. We also produced a standardized written exam summary and conducted a standardized interview about symptoms and quality of life (Patient Assessment of Upper Gastrointestinal (PAGI) Symptoms and Quality of Life). We excluded patients under the age of 18 and patients who did not give their informed consent.

### Esophagogastroduodenoscopy

EGD was performed after a minimum fasting period of 6 h for food and non-clear liquids and 2 h for water. A flexible video esophagogastroduodenoscope (e.g. GIF-HQ190, Olympus Medical Systems, Tokyo, Japan) was used for all examinations. Two doctors and two assisting nurses conducted the EGD. The patient was positioned in the left lateral recovery position, and the examination was performed under sedation with propofol (e.g. Fresenius Kabi Germany GmbH) and under continuous monitoring of patients’ vital parameters. Initially, the oesophagus and stomach were inspected and documented. Biopsies were only taken after EndoFLIP™ measurement. The pylorus was inspected but not yet intubated. Hereafter, the EndoFLIP™ balloon catheter was placed alongside the endoscope into the pylorus.

### EndoFLIP™

The EndoFLIP™ balloon catheter EF-325 N was zeroed in a standardized manner using the calibration chamber of the EndoFLIP™ system. Afterwards, it was lubricated with gel and inserted orally alongside the endoscope. Through endoscopic guidance, the catheter was pushed forward into the stomach and positioned in the pylorus, with the pylorus in the middle of the balloon. The stomach was desufflated, the balloon was filled with 30 ml of saline solution and the correct position was again confirmed endoscopically and through narrowing of the cross-sectional area (CSA) of the balloon. Thirty seconds of steady state was recorded for measurement of pressure, smallest diameter, smallest CSA and distensibility. Using 5-ml inflation steps and 30 s of steady state after each inflation, measurements were performed at 30, 35, 40, 45 and 50 ml filling. Afterwards, the balloon was deflated in 5-ml steps, and an additional 30 s of steady state was recorded after every deflation. After completion of the EndoFLIP™ analysis, the balloon was fully deflated and removed. Finally, the pylorus and the duodenum were endoscopically examined for any pathologies or damage caused by previous treatments, and the examination was completed. We calculated distensibility continuously, as the fraction of the smallest CSA (mm^2^) and pressure (mmHg).

### Gastroparesis Cardinal Symptom Index Score (GCSI), Patient Assessment of Upper Gastrointestinal-Symptoms (PAGI-SYM) and Quality of Life (PAGI-QoL)

The GCSI consists of three subscales of the PAGI-SYM, selected to measure important symptoms related to gastroparesis: nausea/vomiting (three items: nausea, retching and vomiting); post-prandial fullness/early satiety (four items: stomach fullness, inability to finish a normal sized meal, feeling excessively full after meals and loss of appetite); and bloating (two items: bloating and belly visibly larger). The GCSI total score is constructed as the average of the three symptom subscales (10). It can range from 0 to 5, with higher scores reflecting greater symptom severity.

The PAGI-SYM contains 20 items that measure six domains: nausea/vomiting (three items); post-prandial fullness/early satiety (four items); bloating (two items); upper abdominal pain (two items); lower abdominal pain (two items); and heartburn/regurgitation (seven items). Patients rated the severity of each symptom on a 6-point Likert scale from 0 (none) to 5 (very severe) (11).

The PAGI-QoL contains 30 items that cover five subscales: Daily activities (ten items); clothing (two items); diet and food habits (seven items); relationships (three items); and psychological well-being (eight items). Patients rated the severity of each symptom on a 6-point Likert scale from 0 (none) to 5 (very severe). Subscale scores are calculated by averaging across the items within the specific subscale after reversing item scores. The range of scores is 0–5; higher scores indicate a better quality of life (12).

### Data Collection and Statistical Methods

Data was collected retrospectively and includes, among others, age, gender, body mass index, ASA, endoscopic findings, CSA, distensibility and PAGI-QoL score. Continuous variables are presented as means and range. Categorical data are presented as numbers and percentages. The student *T* test—for continuous variables—and the chi square test, for nominal or categorical variables, were used for all bivariate analyses. All tests were two sided, with statistical significance set at *P* ≤ 0.05. Data were analysed by the Stata 11.0 (StataCorp, College Station, TX), SPSS Statistics Version 28 (IBM Corp., Armonk, NY, USA) for Windows (Microsoft Corp, Redmond, WA) and Microsoft Excel Version 2013 for Windows (Microsoft Corp, Redmond, WA).

### Approval

The manuscript was submitted to the local ethics committee, which stated that we are exempt from applying for ethical approval as, under German law, no separate ethics application and statement of ethical approval by the local ethics committee are required for performing purely retrospective clinical studies.

## Results

### Baseline Demographics and Procedural Characteristics

Between November 2021 and March 2022, 66 patients were analysed. The mean age in the patient cohort with non-surgical GP was 49 years and in the patient cohort with post-surgical patients (GP and asymptomatic) 62 years. The differences between non-surgical and post-surgical GP patients were significant. The group of non-surgical GP patients included a higher percentage of women, while the group of post-surgical patients included a higher percentage of men. In addition, the BMI in non-surgical GP patients was significantly higher than that in post-surgical patients. The ASA score was also significantly higher in post-surgical GP patients than in the other two groups, while the CCI was significantly higher in non-surgical GP patients than in the other two groups. Interestingly, asymptomatic post-surgical patients had a higher rate of food retention than non-surgical gastroparetic patients.

### GCSI, PAGI-SYM and PAGI-QoL

**Fig. 2:**
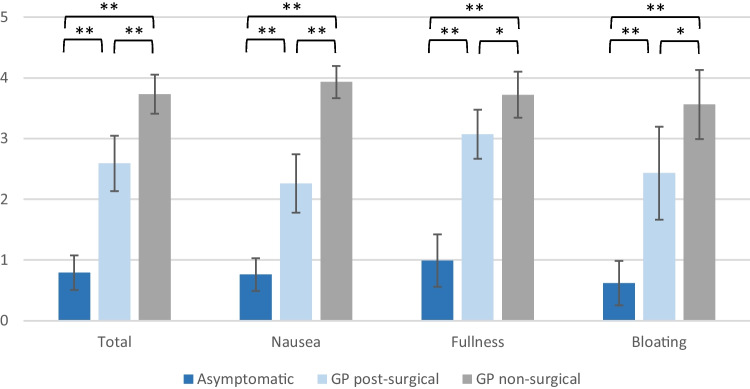
GCSI scores of DGCE and asymptomatic patients, *p < .05, **p < .001

**Fig. 3:**
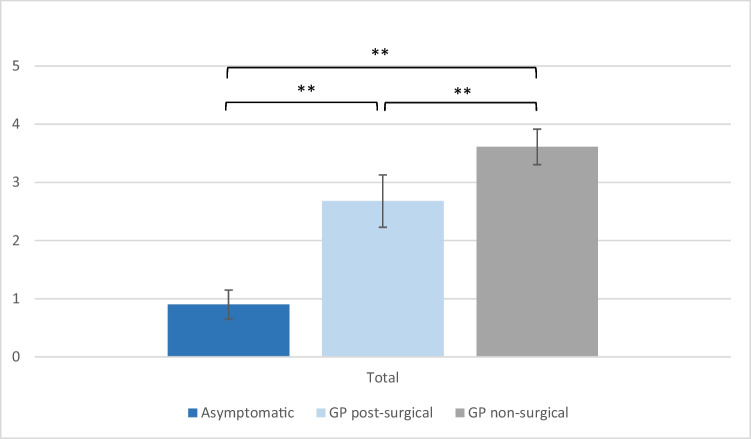
PAGI-SYM scores of DGCE and asymptomatic patients, *p < .05, **p < .01

**Fig. 4:**
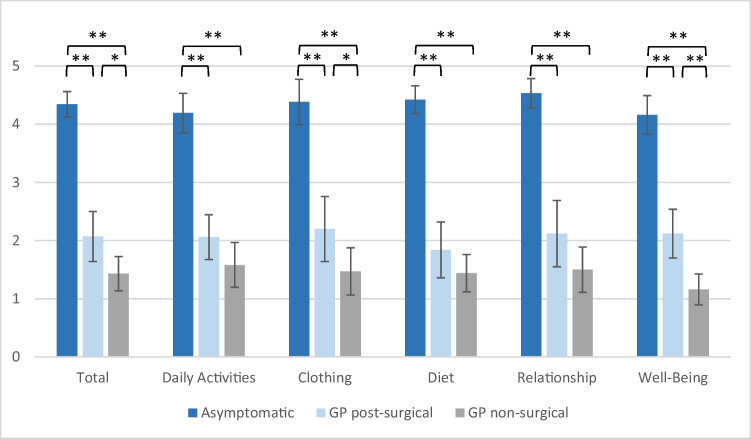
PAGI-QoL scores of DGCE and asymptomatic patients, *p < .05, **p < .01

Data on the GCSI score, PAGI-SYM score and PAGI-QoL score were available in 41 patients with symptoms of gastroparesis (non-surgical and post-surgical) and in 25 patients without symptoms post-surgical. As expected, the PAGI-SYM and GCSI score was significantly reduced in both GP groups in comparison to that in asymptomatic patients (Figs. 2 and 3), whereas the PAGI-QoL showed a significantly higher score in patients without gastroparesis (Table 2, Fig. 4).

**Table 2:** Presenting GCSI, PAGI-SYM and PAGI-QoL in gastroparesis and asymptomatic patients

	**Asymptomatic**	**GP post-surgery**	**GP non-surgical**
GCSI total (mean) [SD]	0.79 (0.57)	2.59 (0.91)	3.73 (0.64)
GCSI nausea (mean) [SD]	0.76 (0.54)	2.26 (0.96)	3.93 (0.53)
GCSI post-prandial fullness (mean) [SD]	0.99 (0.86)	3.07 (0.81)	3.72 (0.76)
GCSI bloating (mean) [SD]	0.62 (0.73)	2.43 (1.53)	3.56 (1.14)
PAGI-SYM total (mean) [SD]	0.90 (0.50)	2.68 (0.90)	3.61 (0.61)
PAGI-QoL total (mean) [SD]	4.34 (0.44)	2.07 (0.86)	1.43 (0.59)
PAGI-QoL daily activities (mean) [SD]	4.19 (0.68)	2.06 (0.77)	1.58 (0.77)
PAGI-QoL clothing (mean) [SD]	4.38 (0.78)	2.20 (1.12)	1.47 (0.81)
PAGI-QoL diet (mean) [SD]	4.42 (0.48)	1.84 (0.96)	1.44 (0.64)
PAGI-QoL relationship (mean) [SD]	4.53 (0.51)	2.12 (1.14)	1.5 (0.78)
PAGI-QoL psychological well-being (mean) [SD]	4.16 (0.66)	2.12 (0.84)	1.16 (0.53)

### EndoFLIP™ Analysis of the Pylorus

EGD and EndoFLIP™ measurements were successfully performed in all patients (*n* = 66, 100%). No severe EndoFLIP™ or EGD-related adverse events occurred, and no patient died because of the procedure. Diameter and distensibility were analysed at 40, 45 and 50 ml balloon filling (Tables 3 and 4); lower balloon fillings provided unreliable results (data not shown). Compared to normal values of pyloric distensibility in healthy individuals, where distensibilities of > 10 mm^2^/mmHg are common,^8^ all our subgroups showed impaired pyloric distensibility. When using a threshold of 10 mm^2^/mmHg and 40 ml balloon filling, 28% (*N* = 5) of non-surgical GP patients, 9% (*N* = 2) of post-surgical GP patients and 8% (*N* = 2) of asymptomatic post-surgical patients showed normal pyloric distensibility.

**Table 3:** Pyloric sphincter distensibility with EndoFLIP™ balloon inflated at 40, 45 and 50 ml

	Balloon volume		
	40 ml	45 ml	50 ml
Non-surgical gastroparesis (mm^2^/mmHg) [SD]	8.16 (3.27)	6.17 (1.67)	4.88 (1.51)
Post-surgical gastroparesis (mm^2^/mmHg) [SD]	5.43 (3.14)	5.13 (2.46)	4.67 (1.91)
Post-surgical asymptomatic (mm^2^/mmHg) [SD]	8.49 (2.57)	7.60 (2.56)	6.27 (2.26)

**Table 4:** Pyloric sphincter diameter with EndoFLIP™ balloon inflated at 40, 45 and 50 ml

	Balloon volume		
	40 ml	45 ml	50 ml
Non-surgical gastroparesis (mm) [SD]	13.2 (2.0)	14.6 (2.1)	15.4 (2.2)
Post-surgical gastroparesis (mm) [SD]	12.1 (2.1)	13.4 (2.2)	14.6 (2.2)
Post-surgical asymptomatic (mm) [SD]	13.4 (1.5)	14.7 (1.8)	15.9 (2.0)

**Fig. 5:**
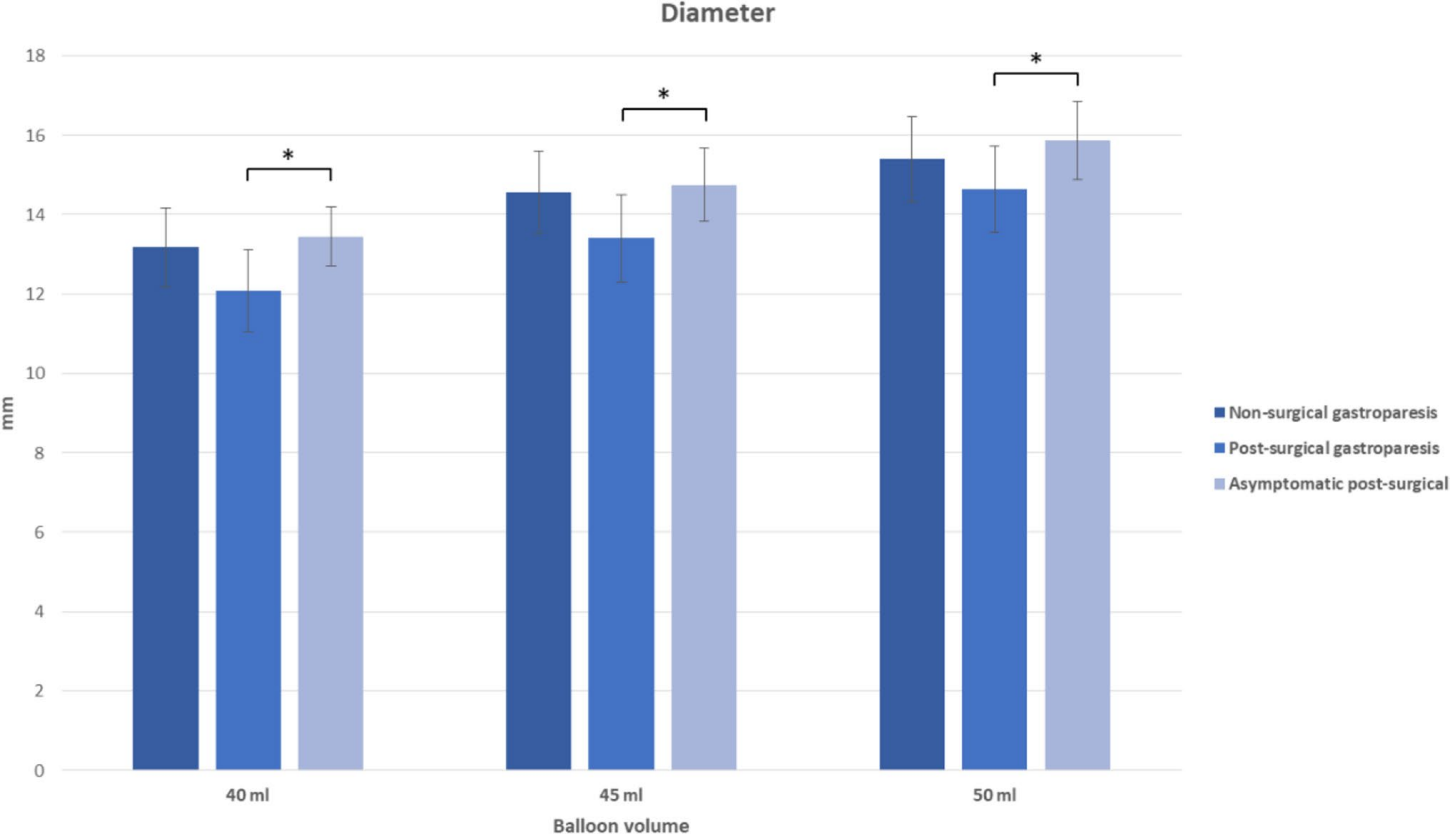
Pyloric sphincter diameter of three subgroups with EndoFLIP™ balloon inflated at 40, 45 and 50 ml, *p < .05

**Fig. 6:**
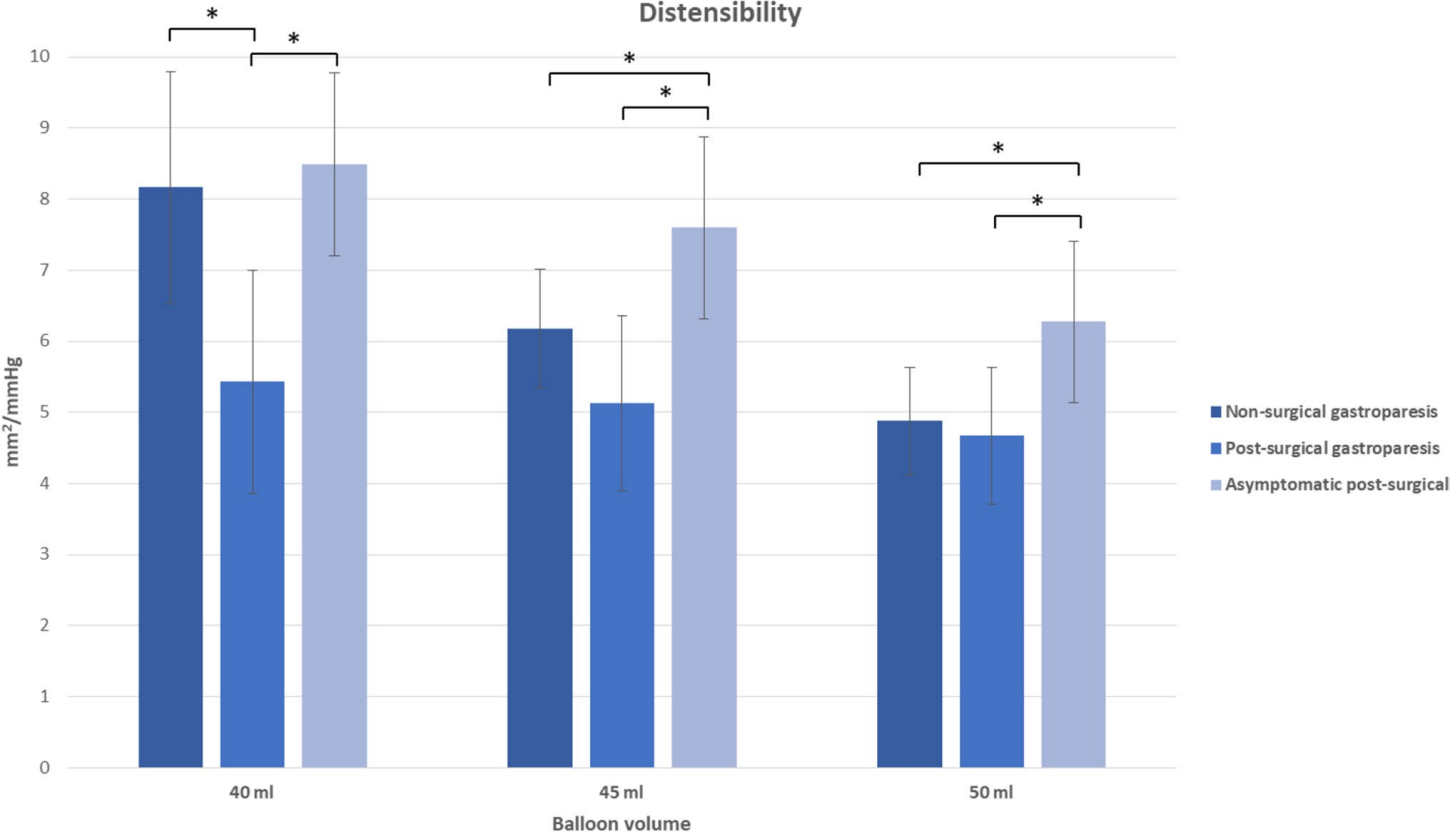
Pyloric sphincter distensibility of three subgroups with EndoFLIP.™ balloon inflated at 40, 45 and 50 ml, *p < .05

Asymptomatic post-surgical patients presented with the highest distensibilities and diameters, while symptomatic post-surgical GP patients presented with the lowest distensibilities and diameters in all balloon fillings. Non-surgical GP patients showed distensibilities and diameters comparable to, but overall higher, than post-surgical GP patients. Significant differences of distensibility between non-surgical and post-surgical GP patients occurred at 40 ml balloon filling. Significant differences of distensibility between non-surgical GP patients and asymptomatic post-surgical patients occurred at 45 and 50 ml balloon filling. Significant differences of distensibility between symptomatic and asymptomatic post-surgical patients occurred at 40, 45 and 50 ml balloon filling (Figs. 5 and 6). Differences and significances were overall clearer for distensibility levels than for CSA, diameters and pressure levels (only partial data shown).

## Discussion

Gastroparesis continues to be a diagnostic and therapeutic challenge for clinicians.^9^ Previous studies demonstrated that, compared to healthy individuals, pyloric distensibility is reduced in patients with gastroparesis^5,8^ as well as in post-surgical patients.^2^ In addition, pyloric distensibility correlates with severity of delayed gastric emptying symptoms.^10^ Studies suggest that pyloric distensibility is a predictive marker of successful therapy of the pylorus with botulinum toxin injection, dilatation or gastric POEM.^5,11,12^ However, the lack of standardization among these studies in terms of probe placement and inflation protocol limits comparability.

The aim of our study was a comparison of pyloric function in different aetiologies of gastroparesis (e.g. post-surgical and non-surgical) and asymptomatic post-surgical patients to find distinguishable differences and common mechanisms. In our retrospective study, we demonstrate that standardized assessment of pyloric function via EndoFLIP™ is easy, safe and feasible in different gastroparesis sub-populations. We establish a standardized protocol to study pyloric distensibility using EndoFLIP™ in the different subgroups. Using this protocol, we can demonstrate common and comparable pyloric distensibilities in all symptomatic sub-populations. Moreover, we were able to demonstrate that, despite post-surgical vagal injury, pyloric distensibility is significantly higher in post-esophagectomy patients than in symptomatic patient sub-populations. Furthermore, asymptomatic post-surgical patients present reduced pyloric distensibility compared to healthy individuals without signs of gastroparesis. We conclude that cut-off levels in gastroparetic patients can identify the patients that profit from pylorus-targeted therapy.

EndoFLIP™ data from GP patients and healthy individuals shows wide variety in existing studies; for example, the mean distensibility in GP patients is described in a range between 4 and 40 mm^2^/mmHg.^6^ The execution of EndoFLIP™ analyses vary between centres. Saadi et al. analyse pyloric function at 30 and 40 ml balloon filling. Snape et al. only use 40 ml filling for analysis,^10^ whereas Malik et al. use 20, 30, 40 and 50 ml balloon filling,^13^ and Gourcerol et al. perform three recordings of every balloon filling.^5^ Some studies lack description of procedure details, making it unclear how EndoFLIP™ measurement was executed. In our centre, we use the analysis technique of Murray et al.^14^ using 30, 35, 40, 45 and 50 ml balloon filling and 30 s of steady state for analysis. We do not intubate or manipulate the pylorus prior to balloon insertion. As our own data suggested different pyloric distensibilities when inflating and deflating the balloon, we did not perform multiple subsequent measurements. We believe that a standardized EndoFLIP™ protocol used in future analyses ensures better homogeneity and comparability of data and propose using the protocol of Murray et al.

Another important aspect of our study is the evaluation of food retention. Gastroparetic, non-surgical patients showed a low rate of food retention in gastroscopy. In comparison, asymptomatic patients after esophagectomy had a high rate of food retention. To our knowledge, no existing study of EndoFLIP™ in gastroparesis described the percentage of food retention in gastroscopy. However, food retention can be an important sign of delayed gastric emptying and in everyday clinical life; it is one potential symptom to consider when suspecting gastroparesis. As it may not correlate with pyloric dysfunction and gastroparesis symptoms in all patient cohorts, an objectifiable technique like gastric emptying scintigraphy is crucial.^15^

As expected, symptom score values were highest in symptomatic patients (PAGI-SYM score, 3.61 in non-surgical GP, 2.68 in post-surgical GP).^16,17^ Non-surgical gastroparesis patients presented with the most severe symptoms and lowest quality of life (PAGI-QoL score 1.43), while post-surgical gastroparesis patients presented with the lowest distensibilities. Many non-surgical gastroparesis patients came to us after multiple pre-treatments and a long disease duration for evaluation of G-POEM, which explains the significantly stronger symptoms when compared to post-surgical gastroparesis patients. As symptoms and quality of life are influenced by multiple factors, e.g. duration of the disease, coping mechanisms and lifestyle, scores may not directly correlate with distensibility.^18^

The strength of this study is its inclusion of a patient cohort of 66 patients in total, divided into three homogenous sub-populations. Most studies so far have only included a small number of patients. Limitations of this study are the retrospective design and the missing evaluation of healthy volunteers. The lack of standardization among studies in terms of probe placement and inflation protocol limits comparability. Furthermore, we acknowledge the heterogeneity of our patient cohorts due to limited patient numbers and the aim to rationally summarize sub-populations. Subsequent studies with higher patient numbers and more precise sub-populations will further expand our knowledge of delayed gastric emptying.

The role of future data should be to further establish cut-off values and normal values of pyloric function in standardized procedures in order to help decide which patient should be treated which way. We propose further prospective studies with different gastroparetic sub-populations and correlate pyloric distensibility with symptoms and therapy success rates.

Our study shows that measurement of the pylorus with EndoFLIP™ is safe and feasible in patients with gastroparesis after upper gastrointestinal surgery as well as in patients without gastroparesis after upper gastrointestinal surgery. Significantly decreased pyloric distensibility in patients with gastroparesis symptoms in comparison to asymptomatic patients enables us to better decide which patient to treat with pylorus-targeted therapy.

In future, EndoFLIP™ analysis could be used as soon as patients develop symptoms and clinical proof of DGE to objectify pyloric dysfunction using cut-off values to accurately plan pyloric interventions such as balloon dilation. Furthermore, as already performed in the esophagogastric junction, intraoperative EndoFLIP™ analysis could help to evaluate pyloric distensibility during upper GI surgery and help decide surgeons when to perform e.g. pyloroplasty during esophagectomy.^19^ As no clinical study investigated this interesting approach yet, this remains an outlook into the future.

## References

[1] Sharma A, Coles M, Parkman HP (2020). Gastroparesis in the 2020s: New Treatments, New Paradigms. Curr Gastroenterol Rep.

[2] Desprez C, Melchior C, Wuestenberghs F (2020). Pyloric distensibility measurement after gastric surgery: Which surgeries are associated with pylorospasm?. Neurogastroenterol Motil.

[3] Camilleri M, Chedid V, Ford AC (2018). Gastroparesis. Nat Rev Dis Primers.

[4] Kwiatek MA, Pandolfino JE, Hirano I (2010). Esophagogastric junction distensibility assessed with an endoscopic functional luminal imaging probe (EndoFLIP). Gastrointest Endosc.

[5] Gourcerol G, Tissier F, Melchior C (2015). Impaired fasting pyloric compliance in gastroparesis and the therapeutic response to pyloric dilatation. Aliment Pharmacol Ther.

[6] Saadi M, Yu D, Malik Z (2018). Pyloric sphincter characteristics using EndoFLIP(®) in gastroparesis. Rev Gastroenterol Mex (Engl Ed).

[7] Abell TL, Camilleri M, Donohoe K (2008). Consensus recommendations for gastric emptying scintigraphy: a joint report of the American Neurogastroenterology and Motility Society and the Society of Nuclear Medicine. J Nucl Med Technol.

[8] Jagtap N, Kalapala R, Reddy DN (2020). Assessment of Pyloric Sphincter Physiology Using Functional Luminal Imaging Probe in Healthy Volunteers. J Neurogastroenterol Motil.

[9] Egboh SC, Abere S (2022). Gastroparesis: A Multidisciplinary Approach to Management. Cureus.

[10] Snape WJ, Lin MS, Agarwal N (2016). Evaluation of the pylorus with concurrent intraluminal pressure and EndoFLIP in patients with nausea and vomiting. Neurogastroenterol Motil.

[11] Desprez C, Melchior C, Wuestenberghs F (2019). Pyloric distensibility measurement predicts symptomatic response to intrapyloric botulinum toxin injection. Gastrointest Endosc.

[12] Jacques J, Pagnon L, Hure F (2019). Peroral endoscopic pyloromyotomy is efficacious and safe for refractory gastroparesis: prospective trial with assessment of pyloric function. Endoscopy.

[13] Malik Z, Sankineni A, Parkman HP (2015). Assessing pyloric sphincter pathophysiology using EndoFLIP in patients with gastroparesis. Neurogastroenterol Motil.

[14] Murray FR, Schindler V, Hente JM (2021). Pyloric dilation with the esophageal functional lumen imaging probe in gastroparesis improves gastric emptying, pyloric distensibility, and symptoms. Gastrointest Endosc.

[15] Sullivan A, Temperley L, Ruban A (2020). Pathophysiology, Aetiology and Treatment of Gastroparesis. Dig Dis Sci.

[16] Revicki DA, Rentz AM, Dubois D (2004). Gastroparesis Cardinal Symptom Index (GCSI): development and validation of a patient reported assessment of severity of gastroparesis symptoms. Qual Life Res.

[17] Rentz AM, Kahrilas P, Stanghellini V (2004). Development and psychometric evaluation of the patient assessment of upper gastrointestinal symptom severity index (PAGI-SYM) in patients with upper gastrointestinal disorders. Qual Life Res.

[18] Revicki DA, Camilleri M, Kuo B (2009). Development and content validity of a gastroparesis cardinal symptom index daily diary. Aliment Pharmacol Ther.

[19] Su B, Dunst C, Gould J (2021). Experience-based expert consensus on the intra-operative usage of the Endoflip impedance planimetry system. Surg Endosc.

